# Supplementary material for: Sugar Substitute Stevia Inhibits Biofilm Formation, Exopolysaccharide Production, and Downregulates the Expression of Streptococcal Genes Involved in Exopolysaccharide Synthesis
Source: Dent J (Basel). 2023 Nov 23;11(12):267. doi: 10.3390/dj11120267 (PMC10742993; doi:10.3390/dj11120267)
Supplement: Supplementary file 1 [file dentistry-11-00267-s001.zip › dentistry-2584336-supplementary.pdf]

Supplementary Table S1. Measurement of pH from the biofilms.

	pH Value			
	S. mutans		*S. gordonii*	
Conc. mg/ml	Sucrose	Stevia	Sucrose	Stevia
0	6.5	6.5	6.6	6.5
1	6.1	6.4	6.2	6.3
5	5.1	5.9	5.7	6.1
10	4.6	5.7	5.1	6.5
25	4.0	5.2	4.8	6.5

Supplementary Table S2. Exopolysaccharide production from the biofilms.

S. mutans

Sucrose (mg/ml)	Mean pellet	SD	Mean SUP*	SD	Stevia (mg/ml)	Mean pellet	SD	Mean SUP	SD
0	100	0	100	0	0	100	0	100	0
1	117.6201035	4.222781	130.994898	9.199604	1	51.58325842	2.74056	124.8304	3.453982
5	112.7734664	3.493724	216.5072279	1.69862	5	40.6365498	0.20307	114.2809	0.911467
10	115.4988914	1.222934	141.7517007	0.120256	10	35.86429927	0.570361	136.9064	12.08894
25	77.50739098	0.096685	117.0493197	5.351403	25	25.6168215	0.62157	128.5617	1.822935

S. gordonii

Sucrose (mg/ml)	Mean pellet	SD	Mean SUP	SD	Stevia (mg/ml)	Mean pellet	SD	Mean SUP	SD
0	100	0	100	0	0	100	0	100	0
1	100.5882353	17.4697	124.8303935	3.453982	1	57.19245543	0.212418	114.3836	2.905918
5	96.47058824	8.318903	114.2808684	0.911467	5	65.95686004	3.245462	91.78082	0
10	85.88235294	1.663781	136.9063772	12.08894	10	44.90938639	1.166556	99.31507	4.843197
25	68.82352941	0.83189	128.5617368	1.822935	25	47.3702354	0.731274	93.83562	0.968639

*SUP=Supernatant
